# Traditional Chinese Medicine for Metabolic Syndrome via TCM Pattern Differentiation: Tongue Diagnosis for Predictor

**DOI:** 10.1155/2016/1971295

**Published:** 2016-05-25

**Authors:** Tsung-Chieh Lee, Lun-Chien Lo, Fang-Chen Wu

**Affiliations:** ^1^Department of Traditional Chinese Medicine, Changhua Christian Hospital, Changhua 500, Taiwan; ^2^Graduate Institute of Statistics and Information Science, National Changhua University of Education, Changhua 500, Taiwan; ^3^Department of Bioindustry Technology, DAYEH University, Changhua 51591, Taiwan

## Abstract

Metabolic syndrome is a morbid condition, which is manifested by central obesity, abnormal glucose tolerance, lipodystrophy, and hypertension. Traditional Chinese medicine (TCM) clarifies that obesity is classified as phlegm-dampness. It is often accompanied with qi stagnation and blood stasis. One hundred and two overweight adults, who did not receive lipid-lowering drugs, were enrolled for analysis. The exclusion criteria were adults having malignancy disease, DM, and renal disease or who were pregnant or lactating. The study was divided into two groups: metabolic syndrome group (MetS) and nonmetabolic syndrome group (nMetS). The modern tongue analysis and heart rate variability devices for data analysis and Council on Nutrition Appetite Questionnaire (CNAQ) for appetite evaluation were used. Obesity patients with metabolic syndrome obviously have lower CNAQ score. The 6 items of CNAQ between two groups have significant difference in variation (*P* < 0.001). The nMetS average was above 28 scores (96%) and the MetS was all in 17–28 scores. The tongue appearance showed that MetS group have white coating different from the nMetS group with white and yellow coating (*P* < 0.05). However the HRV is not different from nMetS group significantly. Our results try to explore the relationship between the TCM pattern, nutrition appetite, and heart rate variability in metabolic syndrome patients.

## 1. Introduction

Obesity is a complex disease, which results from weight gain and is secondary to the positive energy storage. Obesity is induced by environment nutrition [1], shortened sleep [2], drug, virus [3], gene [4–6], and endocrine disorder [7, 8]. It often increases the risk of hyperlipidemia [9], diabetes [10], and hypertension [11]. Study showed that obesity-related hypertension is often complicated to cardiovascular (CV) autonomic dysfunction [12]. It also comments that children obesity can lead to vascular arthrosclerosis. Abdominal obesity drives the progression of multiple cardiometabolic diseases. The high waist circumference has been recognized within the diagnostic criteria to identify individuals with features of the metabolic syndrome. NCEP-ATP III (2001) considered the “obesity epidemic” mainly responsible for the raising prevalence of metabolic syndrome. According to the principle, metabolic syndrome was defined as criteria of waist circumference (men > 102 cm; women > 88 cm); triglycerides ≥ 150 mg/dL; HDL cholesterol (men < 40 mg/dL; women < 50 mg/dL); blood pressure ≥130/≥85 mmHg; and fasting glucose ≥ 110 mg/dL. When 3 of 5 of the listed characteristics are present, a diagnosis of metabolic syndrome can be made. In Taiwan, the Health Promotion Administration, Ministry of Health and Welfare, corrects the waist circumference to ≥90 cm in men and ≥80 cm in women. In the study, we used the same definition. Heart rate variability (HRV) is an indicator of CV autonomic function and has been linked with hypertension and diabetes [13]. According to the American Heart Association, the HF (high frequency) is 0.15~0.4 Hz and related to parasympathetic nerve function. The LF (low frequency) is about 0.04~0.15 Hz and is often referred to as a sympathetic function. VLF (very low frequency) is below 0.04 and related to the sympathetic nerve function. The LF/HF was defined as an index of cardiac sympathetic-parasympathetic balance. There were studies about low HRV with high risk of myocardial infarction. In TCM, the obesity is often regarded as phlegm-dampness. The phlegm-dampness pattern occurs due to more lipid assumption, alcohol, and being exposed to the rain for a long time. It often leads to qi stagnation and blood stasis; it has the characteristics of thick fur and obesity. The TCM pattern is a characteristic profile of signs and symptoms manifested by a group of patients. There are four main diagnostic TCM procedures: inspection, listening and smelling, inquiry, and palpation. Tongue diagnosis plays a key role in the meridians and the conditions of organs. The characteristic of the tongue reflects the organ status and qi-blood imbalance. TCM practitioners observe the tongue appearance such as the tongue body color or tongue coating to determine the pathogenic factors. The color reflects the condition of the yin organs, the blood, and the nutritive qi. Tongue coating is observed from tip to the root and reflects the hot or cold aspects of body [14]. We used the Chinese diagnosis tongue analyzer to classify the TCM pattern. We often thought that people who have more food intake often gain weight easily. To realize the appetite of obesity patient, Council on Nutrition Appetite Questionnaire (CNAQ) [15, 16] was applied. The CNAQ is short and easy to use; it has been used for the adult community or care home residents who are at risk of future weight loss. It also can be used for predicting weight loss in patients with cancer although it does not specify that it is to metabolic syndrome [16].

In this study, we cross-analyzed the effects of autonomic nervous system, appetite score, and the tongue appearance to reveal TCM pattern in obesity adults with metabolic syndrome.

## 2. Materials and Methods

### 2.1. Study Subjects

**Figure 1:**
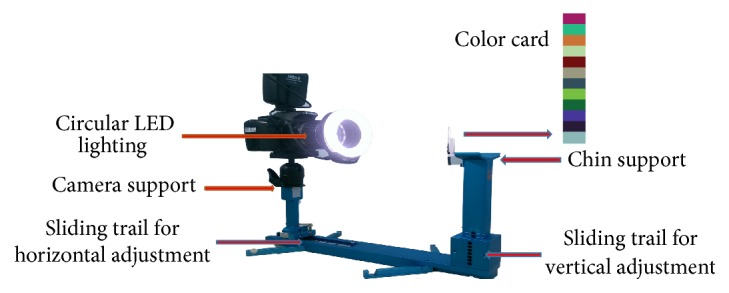
Illustration of tongue image capturing with the ATDS.

We surveyed the overweight patients who visited the Chinese Medicine Department, Changhua Christian Hospital, from January 2013 to December 2015. They were all informed about the study and consent form that needed to be signed. The study was approved by the Changhua Christian Hospital Ethics Committee (IRB number 111111). Informed consent was obtained from the patients before the data was collected. Metabolic syndrome is a clustering of at least three of five of the following medical conditions: abdominal (central) obesity, elevated blood pressure, elevated fasting plasma glucose, high serum triglycerides, and low high-density lipoprotein (HDL) levels. The study group were divided into two groups: metabolic syndrome group (MetS) and nonmetabolic syndrome group (nMetS). In order to simplify the tongue diagnosis, only three typical coatings (thin white, white, and yellow) and three typical tongue colors (pink, red, and dark red) were identified for analysis. The tongue coating and tongue body color were evaluated by the TCM doctor who collected the information with a digital camera (Canon 450D) and color card (Figure 1). We used the autonomic tongue diagnosis system (ATDS) for tongue appearance diagnosis [17].

### 2.2. Inclusion and Exclusion Criteria

The inclusion criteria were patients with body mass index (BMI) > 24 kg/m^2^ of either sex between the age of 18 and 65 years and all of them did not receive lipid-lowering medicines. The following categories of patients were excluded: patients having malignancy disease, DM, and renal disease; patients who cannot complete the research; and pregnant or lactating women.

### 2.3. Sample Size

By the BMI cutoff, there were one hundred and two obesity subjects approached (25 male; 77 female). There were also seven patients (6.8%) who did not complete the study and dropped out. It was a cross-sectional study and 95 patients were enrolled.

### 2.4. Methods and Statistics

A total of 95 patients were enrolled. Participants included were mostly females (*n* = 75). All of the patients were divided into two groups: MetS group (*n* = 45) and nMetS group (*n* = 50). All anthropometric measurements were performed by the same person. Body weight, height, and waist circumference were also measured. Waist circumference was measured between the highest point of iliac crest and lower point of costal margin in the midaxillary line. BMI was calculated as weight divided by height squared in meters (kg/m^2^). Blood pressure was measured after resting using a standard mercury manometer. Venous blood samples for fasting glucose, cholesterol, and triglyceride levels were obtained. We used the modern tongue analysis device for tongue appearance diagnosis and ANS Watch [18] for heart rate variability (HRV). HRV analyzes the frequency domain and time domain of the heartbeat. It is often used as the method to evaluate the sympathetic tone. The intervals of heartbeats were recorded and calculated by Fourier Transform subsequently. We analyzed the HRV by the sitting position and the heart rate monitor (ANS Watch® monitor, Taiwan Scientific Corp.) [18]. R-R intervals were recorded every 5 minutes in a quiet room and data was used to assess HRV. Natural logarithms (ln) of total power (ln⁡TP), very low frequency (ln⁡VLF), low frequency (ln⁡LF), and high frequency (ln⁡HF) were used to evaluate HRV.

**Figure 2:**
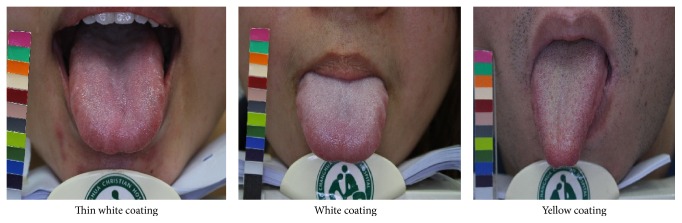
Samples of tongue coating images. Tongue coating: a layer of moss-like material covering the tongue. Thin white coating: a tongue coating through which the underlying tongue surface is faintly visible. White coating: tongue coating white in color. Yellow coating: tongue coating yellow in color.

The TCM pattern identification was the process of overall analysis of clinical data used to determine the location, cause, and nature of a patient's disease; it achieved a diagnosis of a pattern/syndrome, also called pattern differentiation. Chinese medicine patterns were classified by inspection, listening and smelling, inquiry, and palpation. The inspection of the tongue is the examination of the tongue body, and its coating also means tongue appearance. There were many tongue colors in TCM; in order to simplify the tongue appearance, the tongue colors were divided into pink, red, and dark red, and the tongue coatings were divided into thin white, white, and yellow coating (Figure 2). Council on Nutrition Appetite Questionnaire (CNAQ) is an 8-item single-domain questionnaire. Response was using a 5-point (A to E), verbally labeled, Likert-type scale. While lower scores indicate deterioration in appetite, a total score of 28 or less is defined as severe loss of appetite. The range was from 8 (worst) to 40 (best).

### 2.5. Data Analysis

Analysis was performed with the SPSS statistical software package, version SPSS 22.0 (Chicago, IL, USA). Significant differences between groups were analyzed using the chi-square or Fisher's Exact Test for categorical data, and the Mann-Whitney *U* test was used to analyze difference between medians of the two datasets for continuous variable. *P* value less than 0.05 was considered statistically significant.

## 3. Results

The study is carried out by 102 (25 male; 77 female) obesity patients selected from Changhua Christian Hospital. It was a cross-sectional study, and 95 patients were enrolled. Participants included were mostly females (*n* = 75). All of the obesity patients were divided into two groups of MetS group and nMetS group. There was no significant difference between two groups even in body weight, waist circumference, and vessel blood examination (Table 1). The mean age of MetS group was 38.1 ± 8.5 and that of nMetS group was 37.4 ± 10.0 (mean ± SD). The BMI were 28.9 ± 5.9 and 29.1 ± 5.1 kg/m^2^ (mean ± SD) independently.

### 3.1. Characteristics of the Research Participants

**Figure 3:**
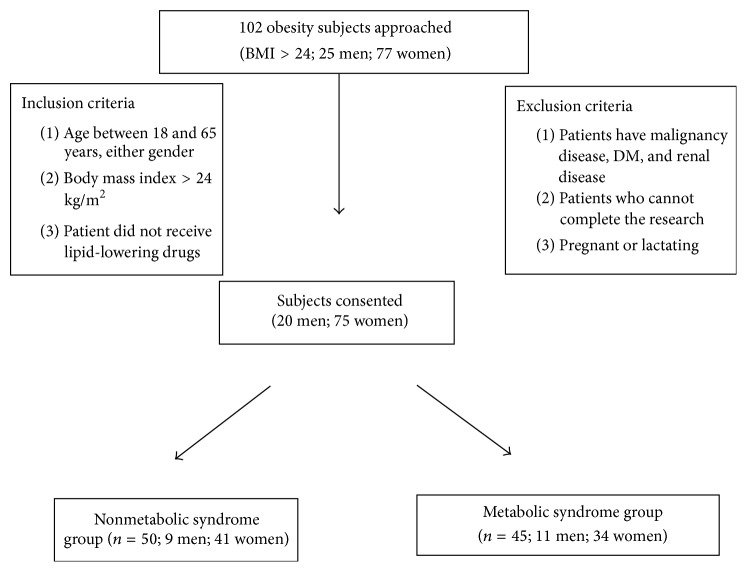
Flow diagram of recruitment of subjects.

Figure 3 shows the flow of recruitment of subjects. Ninety-five patients were included in the study, fifty in the nMetS group and forty-five in MetS group. Baseline characteristics were similar between the two groups even in gender, age, and BMI (Table 1). Elevation of systolic blood pressure (≥130 mmHg) was present in 26.5% and elevation of diastolic blood pressure (≥85 mmHg) was in 14.3% in the MetS group. The hypertriglyceridemia was detected in 28.6% in the MetS group and average is 151.67 mg/dL; the mean HDL was 45.65 mg/dL.

### 3.2. Appetite Assessment

Table 2 showed the CNAQ between the two groups. The sum of the CNAQ scores was different in the efficacy between the two groups (Table 2). The 6 items also have a significant difference in variation (*P* < 0.001). The nMetS average was above 28 scores (96%) and the MetS was all in 17–28 scores (100%).

### 3.3. Tongue Appearance Analysis

In TCM pattern, there was no difference between the two tongue color groups (Table 3). However, the tongue coating in the nMetS group was white coating in about 79.6% and yellow in 20.4%. The MetS group had white coating in 100% and yellow coating in 0%. The difference between the groups is significant (*P* = 0.002).

### 3.4. Heart Rate Variability

Table 4 demonstrates the median and interquartile range values for variables of time and frequency domain between MetS and nMetS groups. In analysis between the two groups, the mean ln⁡VLF (ms^2^/Hz), the mean ln⁡LF, the mean ln⁡HF, and the mean of LF/HF ratio were not different between two groups (*P* > 0.5). Statistical analysis revealed no significant differences between two groups in either gender or basic characteristics.

## 4. Discussion

We aimed to study the obesity patients' lifestyle and Chinese medicine pattern. The Chinese medicine classified the obesity as a phlegm-dampness pattern. In TCM, we often observe the tongue appearance for diagnosis. The tongue appearance means the tongue color and tongue coating. In the study, we found the MetS patients having a well-marked white tongue coating more than thin white coating. Otherwise, the nMetS had white coating and yellow coating. According to the CNAQ, the MetS often has more than 28 scores (96%), but the nMetS overall was under the 28 scores (*P* < 0.001). A total score of 28 or less is defined as “severe loss of appetite” and predicts a weight loss of at least 5% within next 6 months [15]. By the questionnaire analysis, the two groups felt hungry from occasionally to some of the time with no significant difference (*P* = 0.006). The MetS often has three meals a day and the nMetS takes more than three meals a day (*P* = 0.006). We also found that the MetS is often in the mood of neither sad nor happy (73%) and nMetS group often in the mood of happy (62%). As for the satiety, about 80% of nMetS group felt full after eating most of the food. About 46.7% of the MetS group felt full after over half of a plate/meal and 42.2% after most of the food. The obesity results from weight gain secondary to positive energy balance and is often due to more food intake over expenditure. But most of the study is aimed at the content of diet like DASH and diary product. There were journals that discussed the content of food and lacked the observation of dietary habit [19, 20] or exercise. In the past, we found that the metabolic syndrome patients have unhealthy food with high mortality in China [21, 22]. Appetite is a complicated interaction between nervous system and end-organs [23]. We often mean that metabolic syndrome group has more nutrition accumulation than the normal group. HRV measurement is a noninvasive technique that can be a parameter for metabolic syndrome. There were studies showing that impairment in the autonomic nervous syndrome is often related to obesity [24]. In the present study there was no significance between two groups in HRV. Additionally, the frequency of stool passage did not show any difference (1.7 ± 1.0 versus 1.6 ± 1.0). Maybe we learned that the sympathetic tone was not different. The yellow tongue coating often means that it has hot constitutions in TCM that also means that there is high energy. We can hypothesize that the yellow tongue coating patients have better metabolism. HRV can be an index to advanced analysis of the relationship between the yellow tongue coating and the autonomic function. Maybe it can serve us results of obesity patients with nonmetabolic syndrome and having a better sympathetic tone. Especially, since obesity has decreased vagal activity [25], studies showed that the reduction in parasympathetic activity could result in the development of metabolic syndrome [26]. However the present study finds that nondifference, but we did not trace the habit before the metabolic syndrome occurred. There was study where the decreased vagal activity may be caused by the insulin resistance [27]. In this study, the DM patient was excluded, which would lead to the results of nonimpaired vagal activity. We can also hypothesize that the metabolic syndrome restricts the dietary habit. Not all patients with metabolic syndrome have history of hypertension. We should have classified the subgroup analysis.

TCM focuses on qi-blood balance. In normal condition the obstruction of phlegm-dampness can be relaxed by circulation. Tongue color and coating mean that tongue appearance is only one part of that diagnosis in TCM. Tongue color indicates the state of blood. Normal tongue often shows pink or light red. TCM has many constitutions, especially hot and cold patterns [28]. Dark reddish tongue usually indicates excess heat. A red tongue color with a yellow coating may reflect excess heat. Tongue coating indicates the state of the organ, especially the stomach. A thin white tongue coating is a normal state. A white coating often means damp-cold. A yellow tongue coating indicates an interior heat pattern. A type of interior pattern/syndrome with exuberant heat arising when external pathogens enter the internal organs and transforming into heat is mainly manifested by fever with sweating, thirst with intake of fluid, vexation, bitter taste in the mouth, short voiding of reddish urine, a reddened tongue with yellow coating, and rapid surging or rapid string-like pulse. In this study, we found that the MetS patients have white coating, and nMetS individuals have white and yellow coating. The results in this study suggest that tongue coating and body color might be used for metabolic state in obesity interventions.

Obesity is a complex trait, comprised of behavioral, epidemiologic, and molecular/metabolic factors [29]. Physical activity and obesity are closely related [30]. There was a study which revealed that behavior of life like the nutrition and physical activity may influence the obesity [1]. We thought that it might have correlated with the different food or different lifestyle. Study also showed that the time-delayed pattern of eating can induce adiposity and lipid metabolic disorders [31]. Food ingredients act also as antiobesity agents [32]. But hypocaloric intervention did not improve parasympathetic activity and it is often improved by diet intervention plus exercise [33]. These results indicate that metabolic syndrome patients have lower metabolic activity. For this purpose the MetS patients cannot decrease the accumulation of caloric storage. The study also showed that more intake food did not increase the risk of metabolic syndrome.

A limitation of this study is that the differential dietary patterns and career type will lead to the different lifestyles. Patient who work more might reveal different heart rate variability. The advanced research should focus on the cohort study and realize the content of dietary effect or different lifestyles. However, there was no evidence showing that dairy intake is correlated with hypertriglyceridemia [34, 35].

## 5. Conclusions

In conclusion, obese MetS patients have lower CNAQ score, and the HRV is not different from nMetS group significantly. However the tongue appearance showed that MetS group have white coating that is different from the nMetS group with white and yellow coating.

## Figures and Tables

**Table 1:** Descriptive characteristics of the sample.

		Metabolic syndrome				Total (*n* = 95)		*P* value
		No (*n* = 50)		Yes (*n* = 45)				
		*N*	%	*N*	%	*N*	%	
Gender	Female	41	82.0	34	75.6	75	78.9	0.442
	Male	9	18.0	11	24.4	20	21.1	

BMI (kg/m^2^)	<24	10	20.0	6	13.3	16	16.8	0.386
	≥24	40	80.0	39	86.7	79	83.2	

Waist circumference (cm)	Men ≤ 90, women ≤ 80	11	22.4	7	15.9	18	19.4	0.425
	Men > 90, women > 80	38	77.6	37	84.1	75	80.6	

Systolic pressure^*∗*^	<130 mmHg	36	73.5	38	84.4	74	78.7	0.194
	≥130 mmHg	13	26.5	7	15.6	20	21.3	

Diastolic pressure^*∗*^	<85 mmHg	42	85.7	42	93.3	84	89.4	0.321
	≥85 mmHg	7	14.3	3	6.7	10	10.6	

Stool passage habit (per day)	<1	5	10.0	5	11.1	10	10.5	
	≥1	45	90.0	40	88.9	85	89.5	

Cholesterol (mg/dL)	<200	21	42.9	22	48.9	43	45.7	0.558
	≥200	28	57.1	23	51.1	51	54.3	

Triglyceride (mg/dL)	<150	35	71.4	32	71.1	67	71.3	0.973
	≥150	14	28.6	13	28.9	27	28.7	

Chi-square test (categorical variables); ^*∗*^ *P* value using Fisher's Exact Test.

Values given are number of patients (%).

**Table 2:** Council on nutrition appetite questionnaire analysis.

		Metabolic syndrome						*P* value
		No (*n* = 50)		Yes (*n* = 45)		Total (*n* = 95)		
		*N*	%	*N*	%	*N*	%	
(1) My appetite is	Very poor	2	4	0	0	2	2.1	<0.001
	Poor	0	0	1	2.2	1	1.1	
	Average	11	22	38	84.4	49	51.6	
	Good	21	42	5	11.1	26	27.4	
	Very good	16	32	1	2.2	17	17.9	

(2) When I eat, I feel full after	Eating only a few mouthfuls	0	0	3	6.7	3	3.2	<0.001
	Eating about a third of a plate/meal	1	2	2	4.4	3	3.2	
	Eating over half of a plate/meal	7	14	21	46.7	28	29.5	
	Eating most of the food	40	80	19	42.2	59	62.1	
	Hardly ever	2	4	0	0	2	2.1	

(3) I feel hungry	Never	4	8	7	15.6	11	11.6	0.006
	Occasionally	8	16	18	40	26	27.4	
	Some of the time	30	60	19	42.2	49	51.6	
	Most of the time	8	16	1	2.2	9	9.5	

(4) Food tastes	Bad	1	2	2	4.4	3	3.2	<0.001
	Average	2	4	26	57.8	28	29.5	
	Good	34	68	17	37.8	51	53.7	
	Very good	13	26	0	0	13	13.7	

(5) Compared to when I was 50, food tastes	Worse	1	2	15	33.3	16	16.8	<0.001
	Just as good	24	48	24	53.3	48	50.5	
	Better	9	18	4	8.9	13	13.7	
	Much better	16	32	2	4.4	18	18.9	

(6) Normally, I eat	One meal a day	1	2	0	0	1	1.1	0.006
	Two meals a day	7	14	9	20	16	16.8	
	Three meals a day	33	66	36	80	69	72.6	
	More than three meals a day	9	18	0	0	9	9.5	

(7) I feel sick or nauseated when I eat	Often	0	0	2	4.4	2	2.1	0.001
	Sometimes	1	2	9	20	10	10.5	
	Rarely	23	46	24	53.3	47	49.5	
	Never	26	52	10	22.2	36	37.9	

(8) Most of the time my mood is	Sad	1	2	5	11.1	6	6.3	<0.001
	Neither sad nor happy	18	36	33	73.3	51	53.7	
	Happy	28	56	7	15.6	35	36.8	
	Very happy	3	6	0	0	3	3.2	

Total scoring	17–28	2	4	45	100	47	49.5	<0.001
	>28	48	96	0	0	48	50.5	

*P* value using Fisher's Exact Test.

Values given are number of patients (%).

**Table 3:** Tongue color and coating between MetS and nMetS groups.

	Metabolic syndrome					Total (*n* = 95)		*P* value
	Level	No (*n* = 50)		Yes (*n* = 45)				
		*N*	%	*N*	%	*N*	%	
Tongue coating	Thin white	8	16.3	6	13.6	14	15.1	0.002
	White	31	63.3	38	86.4	69	74.2	
	Yellow	10	20.4	0	0.0	10	10.8	

Tongue color	Pink	27	55.1	27	61.4	54	58.1	0.461
	Red	22	44.9	16	36.4	38	40.9	
	Dark red	0	0.0	1	2.3	1	1.1	

*P* value using Fisher's Exact Test.

Values given are number of patients (%).

**Table 4:** The median and interquartile range values for variables of time and frequency domain between MetS and nMetS groups.

	Metabolic syndrome						*P* value
	No			Yes			
	*N*	Median	IQR	*N*	Median	IQR	
ln⁡VLF (ms^2^/Hz)	50	6.2	5.7–6.8	43	6.2	5.6–7.0	0.697
ln⁡LF (ms^2^/Hz)	50	5.5	5.0–6.4	43	5.5	4.8–6.3	0.951
ln⁡HF (ms^2^/Hz)	50	5.4	4.6–5.9	43	5.3	4.5–6.0	0.844
LF/HF	50	1.0	0.9–1.1	43	1.0	0.9–1.2	0.655

*P* value using Mann-Whitney *U* test.

VLF: very low frequency, HF: high frequency, LF: low frequency, and IQR: interquartile range.

## References

[1] Inal S., Canbulat N., Bozkurt G. (2015). The effects of healthy lifestyle behaviors of mothers on obesity in preschool children. *Journal of the Pakistan Medical Association*.

[2] Rangaraj V. R., Knutson K. L. (2016). Association between sleep deficiency and cardiometabolic disease: implications for health disparities. *Sleep Medicine*.

[3] Park S., Park H.-L., Lee S.-Y., Nam J.-H. (2016). Characteristics of adipose tissue macrophages and macrophage-derived insulin-like growth factor-1 in virus-induced obesity. *International Journal of Obesity*.

[4] Mason K., Page L., Balikcioglu P. G. (2014). Screening for hormonal, monogenic, and syndromic disorders in obese infants and children. *Pediatric Annals*.

[5] Zegers D., Hendrickx R., Verrijken A. (2014). Screening for genetic variants in BDNF that contribute to childhood obesity. *Pediatric Obesity*.

[6] Chesi A., Grant S. F. A. (2015). The genetics of pediatric obesity. *Trends in Endocrinology & Metabolism*.

[7] Kirkley A. G., Sargis R. M. (2014). Environmental endocrine disruption of energy metabolism and cardiovascular risk. *Current Diabetes Reports*.

[8] Lizcano F., Guzmán G. (2014). Estrogen deficiency and the origin of obesity during menopause. *BioMed Research International*.

[9] Numasawa Y., Kohsaka S., Miyata H. (2015). Impact of body mass index on in-hospital complications in patients undergoing percutaneous coronary intervention in a Japanese real-world multicenter registry. *PLoS ONE*.

[10] Ridderstråle M., Gudbjörnsdottir S., Eliasson B., Nilsson P. M., Cederholm J. (2006). Obesity and cardiovascular risk factors in type 2 diabetes: results from the Swedish National Diabetes Register. *Journal of Internal Medicine*.

[11] De Nunzio C., Truscelli G., Trucchi A. (2016). Metabolic abnormalities linked to an increased cardiovascular risk are associated with high-grade prostate cancer: a single biopsy cohort analysis. *Prostate Cancer and Prostatic Diseases*.

[12] Zimmet P., Alberti G. K. M. M., Kaufman F. (2007). The metabolic syndrome in children and adolescents-an IDF consensus report. *Pediatric Diabetes*.

[13] Carnethon M. R., Golden S. H., Folsom A. R., Haskell W., Liao D. (2003). Prospective investigation of autonomic nervous system function and the development of type 2 diabetes: the atherosclerosis risk in communities study, 1987–1998. *Circulation*.

[14] Anastasi J. K., Currie L. M., Kim G. H. (2009). Understanding diagnostic reasoning in TCM practice: tongue diagnosis. *Alternative Therapies in Health and Medicine*.

[15] Wilson M.-M., Thomas D. R., Rubenstein L. Z. (2005). Appetite assessment: simple appetite questionnaire predicts weight loss in community-dwelling adults and nursing home residents. *American Journal of Clinical Nutrition*.

[16] Halliday V., Porock D., Arthur A., Manderson C., Wilcock A. (2012). Development and testing of a cancer appetite and symptom questionnaire. *Journal of Human Nutrition and Dietetics*.

[17] Lo L.-C., Chen Y.-F., Chen W.-J., Cheng T.-L., Chiang J. Y. (2012). The study on the agreement between automatic tongue diagnosis system and traditional chinese medicine practitioners. *Evidence-Based Complementary and Alternative Medicine*.

[18] Liang W. C., Yuan J., Sun D. C., Lin M. H. (2009). Changes in physiological parameters induced by indoor simulated driving: effect of lower body exercise at mid-term break. *Sensors*.

[19] Altieri P., Cavazza C., Pasqui F., Morselli A. M., Gambineri A., Pasquali R. (2013). Dietary habits and their relationship with hormones and metabolism in overweight and obese women with polycystic ovary syndrome. *Clinical Endocrinology*.

[20] Fildes A., Mallan K. M., Cooke L. (2015). The relationship between appetite and food preferences in British and Australian children. *International Journal of Behavioral Nutrition and Physical Activity*.

[21] Xu X., Hall J., Byles J., Shi Z. (2015). Dietary pattern is associated with obesity in older people in China: data from China health and nutrition survey (CHNS). *Nutrients*.

[22] Sun J., Buys N., Shen S. (2013). Dietary patterns and cardiovascular disease-related risks in Chinese older adults. *Frontiers in Public Health*.

[23] Bae J., Kim J., Choue R., Lim H. (2015). Fennel (foeniculum vulgare) and fenugreek (trigonella foenum-graecum) tea drinking suppresses subjective short-term appetite in overweight women. *Clinical Nutrition Research*.

[24] Kaufman C. L., Kaiser D. R., Steinberger J., Kelly A. S., Dengel D. R. (2007). Relationships of cardiac autonomic function with metabolic abnormalities in childhood obesity. *Obesity*.

[25] da Silva D. F., Bianchini J. A. A., Antonini V. D. S. (2014). Parasympathetic cardiac activity is associated with cardiorespiratory fitness in overweight and obese adolescents. *Pediatric Cardiology*.

[26] Lambert G. W., Straznicky N. E., Lambert E. A., Dixon J. B., Schlaich M. P. (2010). Sympathetic nervous activation in obesity and the metabolic syndrome—causes, consequences and therapeutic implications. *Pharmacology and Therapeutics*.

[27] Altuncu M. E., Baspinar O., Keskin M. (2012). The use of short-term analysis of heart rate variability to assess autonomic function in obese children and its relationship with metabolic syndrome. *Cardiology Journal*.

[28] Jiang B., Liang X., Chen Y. (2012). Integrating next-generation sequencing and traditional tongue diagnosis to determine tongue coating microbiome. *Scientific Reports*.

[29] Allott E. H., Hursting S. D. (2015). Obesity and cancer: mechanistic insights from transdisciplinary studies. *Endocrine-Related Cancer*.

[30] Chomistek A. K., Shiroma E. J., Lee I. M. (2016). The relationship between time of day of physical activity and obesity in older women. *Journal of Physical Activity and Health*.

[31] Wu T., Guo A., Shu Q. (2015). L-Carnitine intake prevents irregular feeding-induced obesity and lipid metabolism disorder. *Gene*.

[32] Kim K.-H., Park Y. (2011). Food components with anti-obesity effect. *Annual Review of Food Science and Technology*.

[33] Prado D. M., Silva A. G., Trombetta I. C. (2010). Exercise training associated with diet improves heart rate recovery and cardiac autonomic nervous system activity in obese children. *International Journal of Sports Medicine*.

[34] Azadbakht L., Mirmiran P., Esmaillzadeh A., Azizi F. (2005). Dairy consumption is inversely associated with the prevalence of the metabolic syndrome in Tehranian adults. *American Journal of Clinical Nutrition*.

[35] Duffey K. J., Gordon-Larsen P., Steffen L. M., Jacobs D. R., Popkin B. M. (2010). Drinking caloric beverages increases the risk of adverse cardiometabolic outcomes in the coronary artery risk development in young adults (CARDIA) study. *The American Journal of Clinical Nutrition*.

